# Longitudinal patterns of cognitive function and depression: insights from the China Health and Retirement Longitudinal Study

**DOI:** 10.7189/jogh.15.04060

**Published:** 2025-02-28

**Authors:** Lu Yang, Yue Xu, Huashuo Zhao, Ke Wang, Chu Zheng

**Affiliations:** 1 Department of Biostatistics, School of Public Health, Xuzhou Medical University, Xuzhou, Jiangsu, China; 2 Suqian Stomatological Hospital, Suqian, Jiangsu, China; 3 Jiangsu Engineering Research Centre of Biological Data Mining and Healthcare Transformation, Xuzhou Medical University, Xuzhou, Jiangsu, China

## Abstract

**Background:**

With the acceleration of population aging, cognitive impairment and depression have become serious public health challenges in countries around the world. The influencing factors of cognitive trajectory, depression trajectory, and dual trajectories in middle-aged and elderly adults have not been fully studied.

**Methods:**

This study used data from the China Health and Retirement Longitudinal Study database spanning from 2011–2018. Group-based trajectory modelling and group-based dual trajectory modelling were employed to examine different trajectories. Restricted cubic spline and multivariate logistic regression analysis were used to elucidate the relationship between sleep duration and grip strength with these different trajectories. Mediation analysis was conducted to explore the mediating roles of sleep duration and grip strength in the activities of daily living (ADLs) and their impact on these trajectories.

**Results:**

Trajectory analysis identified two longitudinal patterns of cognitive function and depression scores: low and high cognitive group, low and high depression group, respectively, and two states of the dual trajectories of cognition and depression: the stable state group and the state decline group. Sleep duration and grip strength were associated with the cognitive trajectory, depression trajectory and dual trajectories. Sleep duration has an inverted U-shaped relationship with cognitive trajectory. Grip strength was nonlinearly associated with the above trajectories. The mediation effects of sleep duration in the association between ADLs and cognitive, depression and dual trajectories were 3.14, 6.14, and 2.70%. While the mediation effects of grip strength were 7.21, 1.67 and 6.24%, respectively (*P* < 0.05).

**Conclusions:**

Sleep duration and grip strength were not only associated with cognitive, depression, and dual trajectories, but also partially mediate the relationship between ADLs and these trajectories. This study will provide a basis for how to intervene in the cognitive and mental health of middle-aged and elderly adults.

As the demographic shift towards an aging population continues, the prevalence of depression and cognitive impairment presents significant public health concerns. The incidence of depression in middle-aged and elderly populations ranges from 8–20%, with approximately 26% of individuals with cognitive impairment also experiencing depression [1,2]. Furthermore, these two conditions frequently co-occur. In China, a meta-analysis revealed a 28.4% prevalence of depression among the elderly [3], while a cross-sectional study conducted in 2022 reported a cognitive impairment incidence exceeding 20% in the aging community [4]. The prevalence and overlap of these conditions not only impact the well-being and quality of life of individuals but also lead to increased health care expenses, imposing a significant economic strain on individuals, families, and society as a whole [5–7]. Therefore, understanding the potential risk factors for depression and cognitive impairment early on plays a crucial role in delaying and preventing the disease onset in the middle-aged and older adult population.

The relationship between sleep duration, depression and cognitive function has been a focal point of research. Sleep disorders are commonly present in the elderly population and may be accompanied by cognitive decline and deterioration of health conditions [8]. A recent study from the US has shown that actigraphy-measured high sleep fragmentation rather than sleep duration was associated with worse cognition among middle-aged black and white men and women [9]. Other studies have revealed a U-shaped relationship between cognitive impairment and sleep duration, indicating that either excessively long or short sleep duration was associated with a higher risk of cognitive impairment [3,10]. But there are also studies that do not support this association [11,12]. Additionally, several studies have shown a curvilinear relationship between sleep duration and depression, with either excessively long or short sleep duration being related to depression [13,14]. Sleep duration relates to cognitive function via its impact on depression, with sleep duration in turn affecting depression, thereby mediating the relationship between sleep duration and cognitive function [15].

Grip strength as an important indicator of physical function and health status [16,17], is associated with cognitive function and depression. Weak grip strength is notably associated with an increased risk of developing various diseases, adverse health outcomes, disability, dementia, and higher mortality rates [18]. While higher grip strength can reduce the potential for cognitive impairment, which in turn is an important predictor of decreased muscle strength, such as grip strength, indicating a significant reciprocal correlation between grip strength and cognitive function [19]. Additionally, studies have found that grip strength is associated with depression, with older adults who have lower grip strength being more likely to experience depression [20].

The relationship between the activities of daily living (ADLs), cognition function, and depression is also gaining attention from scholars. Widely recognised as an indicator of the activity capacity of the elderly [21], ADLs provide valuable insights into their functional abilities. Research has shown a correlation between ADLs and depression symptoms: depression is a risk factor for disability in ADLs among the elderly, and depression exacerbates disability in ADLs [22,23]. Similarly, cognitive impairment and limited ADLs are risk factors for depression among the elderly, and improving ADLs may help reduce the risk of depression among those with cognitive impairment [24].

However, most existing studies are cross-sectional, focusing on the association at a single time point. Unlike social engagement and diet, which influence cognitive function and depression through various mediating factors [25,26], the associations between sleep duration, grip strength, and ADLs with depression and cognitive trajectories remain unclear. Furthermore, many researchers only focus on one disease, either depression or cognitive impairment, and rarely study both cognitive and depression trajectories and dual trajectories simultaneously. Therefore, there is a need for longitudinal data to explore the relationships between sleep duration, grip strength, ADLs, and the trajectories of cognitive function and depression.

In this study, we utilised data from the China Health and Retirement Longitudinal Study (CHARLS) and employed group-based trajectory modelling (GBTM) and group-based dual trajectory modelling (GBDTM) to explore cognitive trajectory, depression trajectory, and dual trajectories of both. We aimed to investigate the changes in cognitive function and depression over time in middle-aged and elderly adults through longitudinal research and to fully reveal the influencing factors related to different trajectories. Additionally, we investigated the associations between sleep duration and grip strength with these trajectories. Further exploration of the mediating roles of sleep duration and grip strength between ADLs and these trajectories was conducted to provide scientific evidence for the prevention of depression and cognitive impairment in middle-aged and elderly adults.

## METHODS

### Study design and data source

The China Health and Retirement Longitudinal Study (CHARLS) is a comprehensive study that represents Chinese adults aged 45 and above. The primary objective of CHARLS is to elucidate the dynamics of retirement and examine its implications for health, health insurance, and economic welfare. The baseline survey carried out in 2011–2012, encompassed participants across 150 counties in 28 provinces of China, collecting data on socioeconomic status, lifestyle, medication, health status, and functional assessments. In brief, participants in the CHARLS study are selected using a four-stage, stratified, cluster random sampling method. Follow-ups with CHARLS participants are conducted every two years to obtain updated information. The baseline survey (Wave 1) in 2011–2012, the first follow-up survey (Wave 2) in 2013–2014, the second follow-up survey (Wave 3) in 2015–2016, and the third follow-up survey (Wave 4) in 2017–2018 all utilised CHARLS data. The CHARLS survey received ethical approval from the Biomedical Ethics Committee of Peking University, ensuring adherence to international standards for research integrity and participant welfare. For a more detailed description of the research design and sampling procedure, please refer to the cohort study of CHARLS [27].

In this study, we utilised the CHARLS data set (Harmonized, and Wave1–4), and excluded participants who met any of the following conditions:

1. age under 45 years old

2. individuals who did not complete the Center for Epidemiologic Studies Depression Scale (CES-D) and the Mini-Mental State Examination (MMSE)

3. participants with missing data on sleep duration, grip strength, ADLs, and self-rated health status.

Ultimately, 7703 participants were included in the study. The flowchart of participant selection is presented in Figure S1 in the **Online Supplementary Document**.

### Outcome variables

#### Cognitive function

The MMSE is a traditional method for assessing cognitive function, known for its good universality and high accuracy of evaluation [28]. We used the MMSE to assess cognitive function by evaluating four dimensions: orientation (five points), calculation (five points), memory (20 points), and drawing (one point). The orientation dimension involves recalling the year, month, date, day of the week, and season, with one point awarded for each correct answer. The calculation dimension requires the subject to subtract seven from 100 consecutively, five times, with one point awarded for each correct subtraction. The memory dimension includes immediate and delayed recall of word groups, where the interviewer reads 10-word groups randomly to each subject, who must then recall them immediately and again after answering a few questions, with one point awarded for each word group recalled. The drawing dimension requires the subject to draw a specified picture on a blank sheet of paper, with one point awarded for a successful drawing. A higher score indicates better cognitive function of the subject.

#### Depressive symptom

The CES-D scale is recognised for its robust reliability and validity in evaluating depressive symptoms, making it a trusted tool in mental health research [29]. We used the 10-item CES-D depression scoring scale (CES-D10) to rate symptoms of depression [30], which includes statements such as:

1. I was bothered by things that don’t usually bother me

2. I had trouble keeping my mind on what I was doing

3. I felt depressed

4. I felt everything I did was an effort

5. I felt hopeful about the future

6. I felt fearful

7. My sleep was restless

8. I was happy

9. I felt lonely

10. I could not get going.

Each item on the CES-D10 scale is rated from 0–3, with the scores representing the frequency of depressive symptoms experienced:

• 0 indicates rarely or none of the time (<1 day)

• 1 indicates some or a little of the time (1–2 days)

• 2 indicates occasionally or a moderate amount of the time (3–4 days)

• 3 indicates most or all of the time (5–7 days).

The total score on the CES-D10 scale can vary from 0–30, with elevated scores reflecting more pronounced levels of depression. A cut-off score of 10 or higher is employed to identify participants exhibiting significant depressive symptoms. Prior research attests to the commendable reliability and validity of the CES-D10 scale.

### Independent variables

Sleep duration was based on the response to the question, ‘During the past month, how many hours of actual sleep did you get at night (average hours for one night)?’. Grip strength (kg) was measured under the guidance of trained examiners using a hydraulic grip strength meter (TM WL-1000 grip strength meter), with each hand measured twice. The maximum value out of the four measurements was used for analysis, and values of 0 or above 100 kg were considered invalid [31]. The activities of daily living (ADLs) include six items: dressing, bathing, eating, getting in and out of bed, using the toilet, and controlling urination and defecation. Respondents were asked whether they had any difficulty performing these activities with a ‘yes’ or ‘no’ answer. Any limitation in one of these items was classified as a limitation in ADLs.

### Other covariates

According to previous research, age, body mass index (BMI), waist circumference [32], gender, education level [33], smoking status, drinking status [34,35], chronic diseases [36–38], and missing teeth as covariates in the analysis. BMI was calculated as weight (in kg) divided by the square of height (in metres). Education level is divided into ‘below high school’ and ‘high school and above’. Current smoking and drinking status were based on responses to the questions ‘Have you ever smoked’ and ‘Did you drink any alcoholic beverages.’ Chronic diseases included hypertension, diabetes, cancer, chronic lung disease, heart disease, stroke, arthritis, dyslipidaemia, liver disease, kidney disease, digestive system disease, and asthma, assessed based on physician diagnosis or self-report. Tooth loss was defined as all teeth falling out.

### Statistical analysis

#### Trajectories analysis

This study employed GBTM to analyse cognitive, and depression trajectories, and GBDTM to analyse dual trajectories. The GBTM and GBDTM methods allow for trajectory analysis of each variable, the establishment of trajectory models, and then visualisation of the relationships between trajectories through the pairwise association probabilities between different variable trajectories [39]. To identify the model with the optimal number of cognitive and depression trajectories, we determined the best-fitting model based on the Bayesian Information Criterion (BIC) and average posterior probability. Considering the sample size, BIC introduces penalty items related to the number of model parameters, which can effectively prevent the model from being complicated due to the high precision. The smaller the absolute value of BIC, the better the model fitting. As the average posterior probability is closer to one, the better. These criteria aid in selecting the model that most appropriately represents the underlying data patterns while balancing model complexity.

#### Correlation and mediation analysis

Continuous variables were described as mean ± standard deviation (SD), and group comparison was performed using two independent sample *t*-test. Categorical variables were expressed as frequency and percentage (n (%)), and group comparison was performed using the χ^2^ test.

Restricted cubic spline analysis was employed to clarify the relationship between sleep duration, grip strength and different trajectories. Sleep duration and grip strength were further categorised, and multivariate logistic regression analysis was used to explore the association between sleep duration, grip strength and the trajectories, adjusting for other covariates. Odds ratio (OR) and its 95% confidence interval (CI) were reported.

Subsequently, mediation analysis was conducted to explore the mediation effects of sleep duration and grip strength in the relationship between ADLs and different trajectories. The bootstrapped method was used to generate the corresponding 95% CIs of the path coefficients (1000 iterations).

#### Subgroups and sensitivity analysis

Considering that the cognition and depression trajectories likely differed by distinct levels of covariates, we conducted several subgroup analyses to examine our findings' robustness and potential variation. Specifically, we repeated the study in sub-studies stratified by gender (male or female) and age (<65 or ≥65 years old). Furthermore, we conducted a sensitivity analysis on individuals deleted due to missing covariates to validate the robustness of the model.

#### Statistical software and packages

The procedure Proc Traj was used to analyse the cognitive trajectory and depression trajectory over time in SAS, version 9.4 (Cary, USA, 2023). All other analyses were implemented under the *R*, version 4.2.2 (R Core Team, Vienna, Austria, 2022) software computing environment, the glmnet package (version 4.1-4) was applied to conduct the Logistic regression model, the lcmm package (version 2.1.0) was applied to analyse the dual trajectories of cognition and depression, the rms package (version 6.8-0) was applied to conduct restricted cubic spline analysis, the ggplot2 package (version 3.4.2) was used to graph. Finally, the mediation package (version 4.5.0) and the lavaan package (version 0.6-17) were applied to conduct the mediation analysis. And all *P*-values <0.05 (two-sided) were considered statistically significant.

## RESULTS

### Baseline characteristics

The average age of the 7703 participants was 57.70 (SD = 8.15) years, with 3617 males (46.96%), and 831 (10.80%) individuals having an education of high school or above. The four waves cognitive function scores were 14.42 (SD = 5.54), 14.62 (SD = 5.54), 13.99 (SD = 5.74) and 12.49 (SD = 7.02); and depressive symptom scores were 8.27 (SD = 6.23), 7.83 (SD = 5.74), 8.10 (SD = 6.40) and 8.82 (SD = 6.55). There were 1072 (13.91%) participants with limited ADLs, 3015 (39.14%) individuals drinking, 3010 (39.08%) individuals smoking, 1861 (24.16%) individuals suffering from hypertension, while diabetes was 443 (5.75%), cancer in 50 (0.65%), chronic lung diseases in 687 (8.92%), heart diseases in 871 (11.31%), stroke in 147 (1.91%), arthritis in 2639 (34.26%), dyslipidaemia in 745 (9.67%), liver diseases in 262 (3.40%), kidney diseases in 418 (5.43%), digestive system diseases in 1739 (22.58%), asthma in 327 (4.25%), tooth loss in 528 (6.85%), with a mean BMI of 23.57 (SD = 3.61) kg/m^2^, average grip strength of 32.47 (SD = 10.55) kg, and mean sleep duration of 6.37 (SD = 1.84) hours.

### Trajectory models of cognitive function and depression

**Figure 1:**
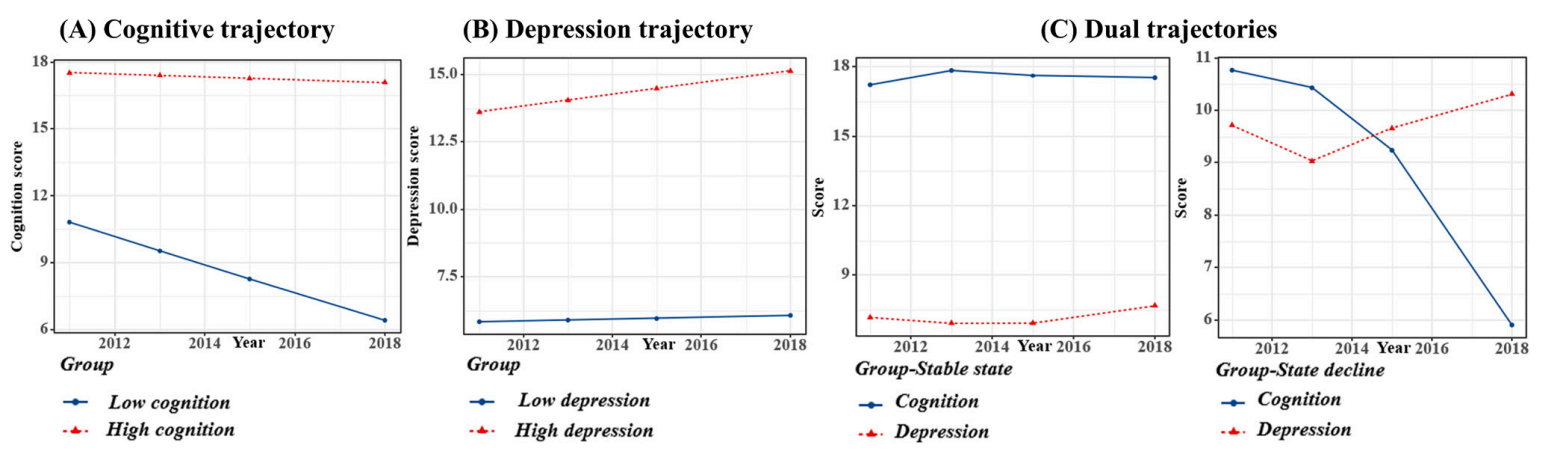
Cognition trajectory (**Panel A**), depression trajectory (**Panel B**), and dual trajectories (**Panel C**) of cognition and depression.

We identified two longitudinal patterns of cognitive function low (n = 4614, 59.90%) and high cognitive group (n = 3089, 40.10%) (**Figure 1**, Panel A), with BIC being -92 432 and the average posterior probability of the two groups was 94.86% and 96.49%, respectively. Similarly, depression was also divided into low (n = 5548, 72.02%) and high depression groups (n = 2155, 27.98%) (**Figure 1**, Panel B), with BIC being -93 696.2 and the average posterior probability of t the two groups was 95.20% and 90.38%. The dual trajectories of cognition and depression: the high cognitive-low depression trajectory, named the stable state group (n = 4369, 56.72%), and the trajectory of declining cognitive function and increasing depression scores, named the state decline group (n = 3334, 43.28%) as shown in **Figure 1**, Panel C. The Bayesian Information Criterion (BIC) of dual trajectories was 374 055.5, the average posterior probability for the stable state group was 90.40%, and for the state decline group was 89.01%.

### Baseline characteristics of different trajectory groups

The foundational characteristics of participants across various trajectory groups, offering a detailed snapshot of the baseline demographics and health statuses pertinent to the study as shown in **Table 1**. Compared to participants with high cognitive function, low depression, and stable state groups, those in the low cognitive, high depression, and state decline groups tended to be older, with grip strength reduction, lower education levels, missing teeth, restricted activities, and shorter sleep duration. The low cognitive group and the high depression group were more likely to occur in females.

**Table 1:** Baseline characteristics of different trajectory groups

Variables	Cognitive trajectory			Depression trajectory			Dual trajectories		
	**Low (n = 3089)**	**High (n = 4614)**	***P-*value**	**Low (n = 5548)**	**High (n = 2155)**	***P-*value**	**Stable state (n = 4369)**	**State decline (n = 3334)**	***P-*value**
Age, years	60.4 ± 8.2	55.9 ± 7.6	<0.001	57.5 ± 8.2	58.1 ± 7.9	0.006	55.7 ± 7.6	60.3 ± 8.2	<0.001
BMI, kg/m^2^	23.0 ± 3.6	24.0 ± 3.6	<0.001	23.7 ± 3.6	23.2 ± 3.8	<0.001	24.0 ± 3.6	23.0 ± 3.6	<0.001
Waist, cm	84.1 ± 10.1	86.4 ± 9.9	<0.001	86.0 ± 9.9	84.3 ± 10.4	<0.001	86.5 ± 9.9	84.2 ± 10.0	<0.001
Grip strength, kg	28.7 ± 9.4	35.0 ± 10.5	<0.001	33.8 ± 10.4	29.0 ± 10.1	<0.001	35.1 ± 10.5	29.0 ± 9.5	<0.001
Male, n (%)	1077 (34.9)	2540 (55.0)	<0.001	2625 (47.3)	1461 (67.8)	<0.001	1943 (44.9)	2123 (63.7)	<0.001
Education, n (%)									
*Under high school*	3069 (99.4)	3803 (82.4)	<0.001	4827 (87.0)	2045 (94.9)	<0.001	3570 (81.7)	3302 (99.0)	<0.001
*High school and above*	20 (0.6)	811 (17.6)		721 (13.0)	110 (5.1)		799 (18.3)	32 (1.0)	
Drinking, n (%)	1059 (34.3)	1956 (42.4)	<0.001	2318 (41.8)	697 (32.3)	<0.001	1850 (42.3)	1165 (34.9)	<0.001
Smoking, n (%)	1033 (33.4)	1977 (42.8)	<0.001	2329 (42.0)	981 (31.6)	<0.001	1860 (42.6)	1150 (34.5)	<0.001
Hypertension, n (%)	753 (24.4)	1108 (24.0)	0.715	1283 (23.1)	578 (26.8)	0.001	1043 (23.9)	818 (24.5)	0.501
Diabetes, n (%)	148 (4.8)	295 (6.4)	0.003	303 (5.5)	140 (6.5)	0.080	276 (6.3)	167 (5.0)	0.015
Cancer, n (%)	21 (0.7)	29 (0.6)	0.783	35 (0.6)	15 (0.7)	0.749	29 (0.7)	21 (0.6)	0.854
Lung, n (%)	304 (9.8)	383 (8.3)	0.020	419 (7.6)	268 (12.4)	<0.001	361 (8.3)	326 (9.8)	0.021
Heart, n (%)	317 (10.3)	554 (12.0)	0.018	512 (9.2)	359 (16.7)	<0.001	527 (12.1)	344 (10.3)	0.017
Stroke, n (%)	71 (2.3)	76 (1.6)	0.041	91 (1.6)	56 (2.6)	0.006	71 (1.6)	76 (2.3)	0.038
Arthritis, n (%)	1233 (39.9)	1406 (30.5)	<0.001	1525 (27.5)	1114 (51.7)	<0.001	1324 (30.3)	1315 (39.4)	<0.001
Dyslipidaemia, n (%)	217 (7.0)	528 (11.4)	<0.001	504 (9.1)	241 (11.2)	0.005	501 (11.5)	244 (7.3)	<0.001
Liver, n (%)	86 (2.8)	176 (3.8)	0.014	165 (3.0)	97 (4.5)	0.001	165 (3.8)	97 (2.9)	0.037
Kidney, n (%)	165 (5.3)	253 (5.5)	0.788	223 (4.0)	195 (9.0)	<0.001	234 (5.4)	184 (5.5)	0.754
Stomach, n (%)	737 (23.9)	1002 (21.7)	0.028	1009 (18.2)	730 (33.9)	<0.001	949 (21.7)	790 (23.7)	0.040
Asthma, n (%)	136 (4.4)	191 (4.1)	0.575	178 (3.2)	149 (6.9)	<0.001	181 (4.1)	146 (4.4)	0.610
Tooth loss, n (%)	303 (9.8)	225 (4.9)	<0.001	348 (6.3)	180 (8.4)	0.001	202 (4.6)	326 (9.8)	<0.001
ADLs, n (%)	622 (20.1)	450 (9.8)	<0.001	484 (8.7)	588 (27.3)	<0.001	424 (9.7)	648 (19.4)	<0.001
Sleep duration, hours	6.2 ± 2.1	6.5 ± 1.6	<0.001	6.6 ± 1.7	5.7 ± 2.0	<0.001	6.5 ± 1.6	6.2 ± 2.1	<0.001

ADLs – activities of daily living, BMI – body mass index

### The association between sleep duration and different trajectories

**Figure 2:**
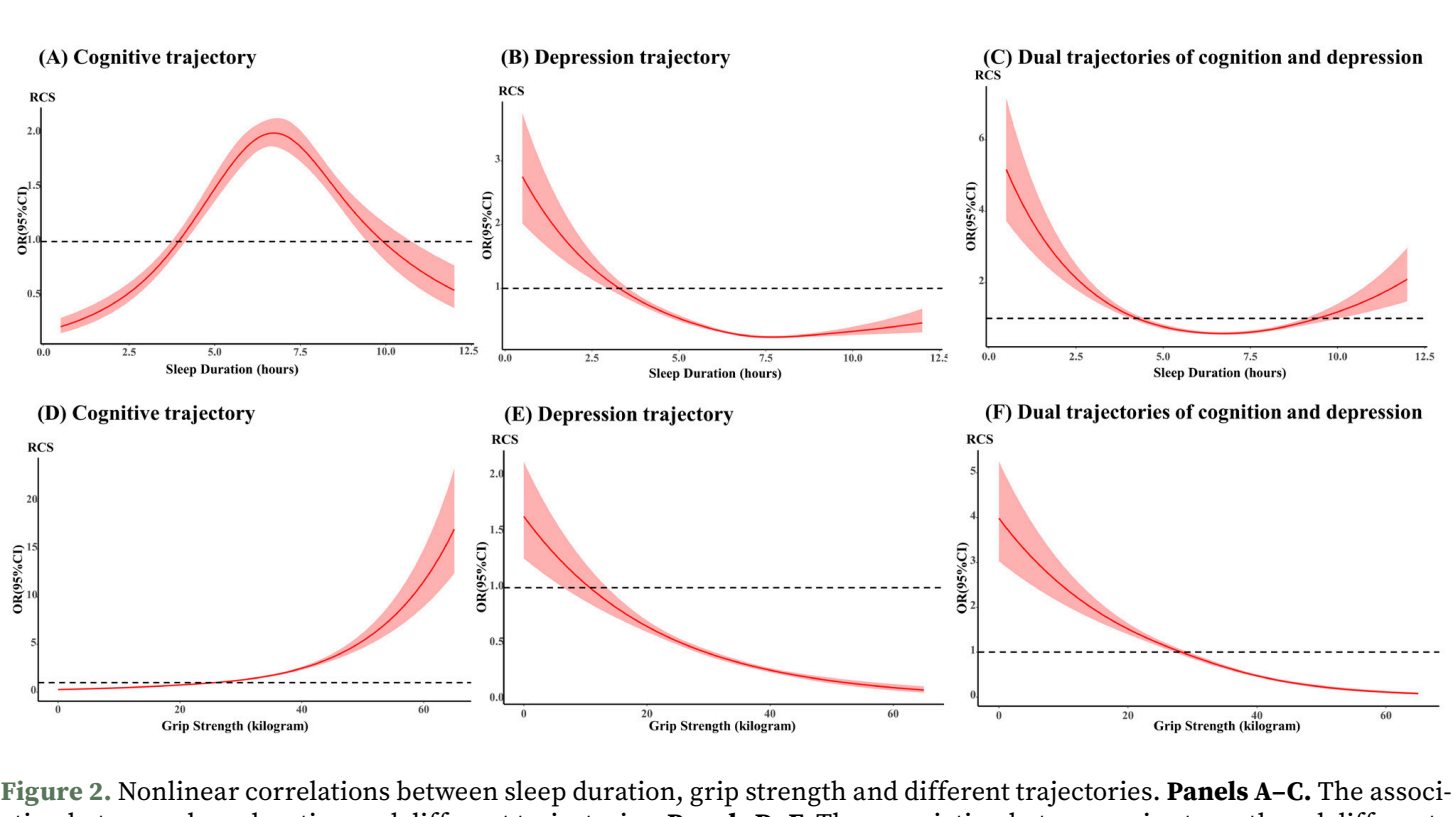
Nonlinear correlations between sleep duration, grip strength and different trajectories. **Panels A–C.** The association between sleep duration and different trajectories. **Panels D–F.** The association between grip strength and different trajectories. OR reflects the strength of the association, and OR>1 indicates that the risk of the trajectory increases due to sleep duration (grip strength), and there is a positive correlation between sleep duration (grip strength) and the trajectory; OR<1 indicates that the risk of disease trajectory decreases due to sleep duration (grip strength), and there is a negative correlation between sleep duration (grip strength) and trajectory. CI – confidence interval. OR – odds ratio.

**Figure 3:**
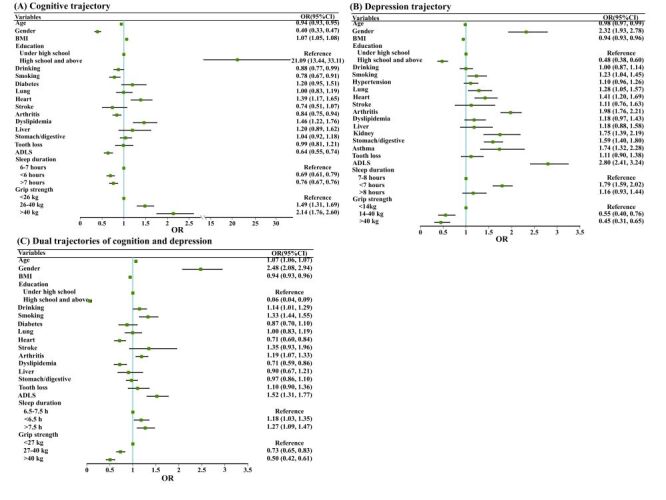
Correlation between sleep duration, grip strength and cognitive trajectory (**Panel A**), depression trajectory (**Panel B**), and dual trajectories of cognition and depression (**Panel C**). OR>1 indicates that the risk of the trajectory increases due to sleep duration (grip strength), and there is a positive correlation between sleep duration (grip strength) and the trajectory; otherwise, there is a negative correlation between sleep duration (grip strength) and trajectory. CI – confidence interval, OR – odds ratio.

Sleep duration was associated with the cognitive function trajectory in an inverted U-shaped relationship (**Figure 2**, Panel A). Taking 6–7 hours of sleep as a reference, participants with less than six hours and more than seven hours of sleep have a risk of high cognitive trajectory of 0.69 (95% CI = 0.61–0.79) and 0.76 (95% CI = 0.67–0.76) times, respectively (**Figure 3**, Panel A). Sleep duration was correlated with depression trajectory and dual trajectories in a U-shaped relationship (**Figure 2**, Panels B–C). The risks of high depression trajectory in participants who slept less than seven hours and more than eight hours were 1.79 (95% CI = 1.59–2.02) and 1.16 (95% CI = 0.93–1.44) times to participants who slept 7–8 hours, respectively (**Figure 3**, Panel B). Similarly, participants with less than 6.5 hours and more than 7.5 hours of sleep had higher risks of low cognitive-high depression trajectory than participants who slept 6.5–7.5 hours, with OR being 1.28 (95% CI = 1.03–1.35) and 1.16 (95% CI = 1.09–1.27), respectively (**Figure 3**, Panel C).

### The relationship between grip strength and different trajectories

The grip strength was nonlinear associated with the cognitive trajectory, depression trajectory and dual trajectories (**Figure 2**, Panels D–F). Compared to participants with the grip strength of less than 26 kg, in participants with grip strengths of 26–40 kg and >40 kg was more suffering from were high cognitive trajectory, with OR being 1.49 (95% CI = 1.31–1.69) and 2.14 (95% CI = 1.49–2.60), respectively (**Figure 3**, Panel A). The risks of high depression trajectory in participants with grip strengths of 14–40 kg and >40 kg were 0.55 (95% CI = 0.40–0.76) and 0.45 (95% CI = 0.31–0.65) times to those with a grip strength of less than 14 kg, respectively (**Figure 3**, Panel B). Compared to participants with a grip strength of less than 27 kg, those with grip strengths of 27–40 kg and >40 kg had 0.73 (95% CI = 0.65–0.83) and 0.50 (95% CI = 0.42–0.61) times the risk of a low cognitive-high depression trajectory, respectively (**Figure 3**, Panel C).

### The association between sleep duration, grip strength and different trajectories in different subgroups

The results revealed differences in the associations between sleep duration, grip strength and different trajectories in different subgroups (**Table 2**). The association between male participants' grip strength and high cognitive trajectory was higher than that of females (2.55 (95% CI = 1.86–3.50) vs. 1.46 (95% CI = 0.95–2.24)), while the associations between female participants' sleep duration and high depression trajectory and state decline trajectory were higher than that of males (1.84 (95% CI = 1.58–2.13) vs. 1.72 (95% CI = 1.42–3.41), 1.36 (95% CI = 1.11–1.66) vs. 1.18 (95% CI = 0.94–1.47)). The correlation between grip strength and high cognitive trajectory in participants under 65 years old was higher than that in participants over 65 years old (2.73 (95% CI = 2.21–3.37) vs. 2.54 (95% CI = 1.62–3.99)), while the correlation between sleep duration and state decline trajectory was less than that in participants over 65 years old (1.19 (95% CI = 1.01–1.39) vs. 1.62 (95% CI = 1.13–2.32)).

**Table 2:** The association between sleep duration, grip strength and different trajectories in different subgroups

Subgroup	Cognitive trajectory		Depression trajectory		Dual trajectories	
	**Variables**	**OR (95% CI)**	**Variables**	**OR (95% CI)**	**Variables**	**OR (95% CI)**
**Male**	**Sleep duration**		**Sleep duration**		**Sleep duration**	
	6–7 h	Reference	7–8 h	Reference	6.5–7.5 h	Reference
	<6 h	0.72 (0.59–0.88)	<7 h	1.72 (1.42–3.41)	<6.5 h	1.15 (0.94–1.42)
	>7 h	0.78 (0.65–0.94)	>8 h	1.13 (0.78–1.63)	>7.5 h	1.18 (0.94–1.47)
	**Grip strength**		**Grip strength**		**Grip strength**	
	<26 kg	Reference	<14 kg	Reference	<27 kg	Reference
	26–40 kg	1.62 (1.21–2.18)	14–40 kg	0.74 (0.36–1.55)	27–40 kg	0.79 (0.60–1.04)
	>40 kg	2.55 (1.86–3.50)	>40 kg	0.58 (0.27–1.22)	>40 kg	0.50 (0.37–0.66)
**Female**	**Sleep duration**		**Sleep duration**		**Sleep duration**	
	6–7 h	Reference	7–8 h	Reference	6.5–7.5 h	Reference
	<6 h	0.67 (0.57–0.79)	<7 h	1.84 (1.58–2.13)	<6.5 h	1.19 (0.98–1.43)
	>7 h	0.74 (0.62–0.97)	>8 h	1.16 (0.88–1.53)	>7.5 h	1.36 (1.11–1.66)
	**Grip strength**		**Grip strength**		**Grip strength**	
	<26 kg	Reference	<14 kg	Reference	<27 kg	Reference
	26–40 kg	1.43 (1.24–1.66)	14–40 kg	0.51 (0.35–0.73)	27–40 kg	0.72 (0.62–0.83)
	>40 kg	1.46 (0.95–2.24)	>40 kg	0.45 (0.26–0.79)	>40 kg	0.71 (0.47–1.08)
**<65 y**	**Sleep duration**		**Sleep duration**		**Sleep duration**	
	6–7 h	Reference	7–8 h	Reference	6.5–7.5 h	Reference
	<6 h	0.71 (0.62–0.82)	<7 h	1.76 (1.54–2.01)	<6.5 h	1.19 (1.03–1.38)
	>7 h	0.83 (0.72–0.95)	>8 h	1.29 (1.01–1.65)	>7.5 h	1.19 (1.01–1.39)
	**Grip strength**		**Grip strength**		**Grip strength**	
	<26 kg	Reference	<14 kg	Reference	<27 kg	Reference
	26–40 kg	1.59 (1.39–1.84)	14–40 kg	0.55 (0.37–0.82)	27–40 kg	0.66 (0.58–0.76)
	>40 kg	2.73 (2.21–3.37)	>40 kg	0.44 (0.29–0.68)	>40 kg	0.39 (0.32–0.48)
**≥65 y**	**Sleep duration**		**Sleep duration**		**Sleep duration**	
	6–7 h	Reference	7–8 h	Reference	6.5–7.5 h	Reference
	<6 h	0.56 (0.42–0.74)	<7 h	1.83 (1.40–2.39)	<6.5 h	1.32 (0.95–1.84)
	>7 h	0.59 (0.44–0.79)	>8 h	0.77 (0.46–1.29)	>7.5 h	1.62 (1.13–2.32)
	**Grip strength**		**Grip strength**		**Grip strength**	
	<26 kg	Reference	<14 kg	Reference	<27 kg	Reference
	26–40 kg	1.81 (1.36–2.40)	14–40 kg	0.56 (0.32–0.98)	27–40 kg	0.62 (0.47–0.83)
	>40 kg	2.54 (1.62–3.99)	>40 kg	0.64 (0.31–1.28)	>40 kg	0.44 (0.28–0.69)

CI – confidence interval, OR – odds ratio

### Subgroup and sensitivity analysis

Subgroup analyses were conducted among different covariate levels. The data are presented in Figure S2–S3 in the **Online Supplementary Document**. We discovered that different subgroups exhibit trajectory classification similar to the full population. However, there were differences in trajectory scores between genders. The scores of cognitive trajectories of men were higher than those of women, while the scores of depression trajectories were lower than those of women. The data are presented in Figure S2 in the **Online Supplementary Document**. Moreover, it was shown that ≥65 years old experienced a faster decline in cognitive function. The data are presented in Figure S3 in the **Online Supplementary Document**. In addition, we identified that the trajectories of cognition and depression still existed if analysing include participants with missing covariates, indicating that our models were robust. The data are presented in Figure S4 in the **Online Supplementary Document**.

### The mediation effects of sleep duration and grip strength in the association between ADLs and different trajectories

The study explored the mediating roles of sleep duration and grip strength in the association between ADLs and different trajectories. The mediation effects of sleep duration in the association between ADLs with cognitive trajectory, depression trajectory, and dual trajectories were 3.14, 6.14, and 2.70%, respectively (*P* < 0.05). The data are presented in Figure S5 (Panels A–C) in the **Online Supplementary Document**. The mediation effects of grip strength in the association between ADLs with cognitive trajectory, depression trajectory, and dual trajectories were 7.21, 1.67, and 6.24%, respectively (*P* < 0.05). The data are presented in Figure S5 (Panels D–F) in the **Online Supplementary Document**.

## DISCUSSION

### Summary of our work

In this longitudinal analysis from CHARLS with 7703 participants, we explored the changes in cognitive function and depression over time in middle-aged and elderly adults. Low and high cognitive group, low and high depression group, and two states of the dual trajectories of cognition and depression were identified. Sleep duration and grip strength were associated with different trajectories. Sleep duration has an inverted U-shaped relationship with cognitive trajectory, while grip strength was nonlinearly associated with the above trajectories. In addition, we indicated that sleep duration and grip strength played partial mediating roles between ADLs and different trajectories.

### Comparison with previous studies

#### Cognitive, depression and dual trajectories

In this work, we observed a correlation between cognitive and depression trajectories. Through dual trajectory analysis, participants were divided into two categories: one group was in a stable state of high cognition and low depression, and the other group was in a state of decreased cognitive function and increased depression scores. It was found that patients with depression often have cognitive function issues [40], and there is a broad overlap between them [41]. Participants with worsening depressive symptoms experienced the fastest decline in cognitive ability, while those with alleviating symptoms did not show a decline [42]. This aligns with the two states of our dual trajectory, which may serve as evidence that depression and cognitive impairment are comorbid. These studies highlight the complexity of the relationship between depression and cognitive function. According to previous research, the classification and shape of cognitive trajectories are not consistent [43,44]. The reason for the inconsistency with our research is that we used different population and trajectory models. Moreover, various subgroup and sensitivity analyses showed similar trajectory grouping indicating the robustness of our models.

In our study, women were more likely to present with low cognition or high depressive status, which is consistent with the results of a study based on the Global Burden of Diseases, Injuries, and Risk Factors Study (GBD) [45]. A Chinese Longitudinal Healthy Longevity Survey (CLHLS) study found women had an overall more serious cognitive decline than men, has been consistent with our research [44]. This difference may be due to differences in brain structure and neurotransmitters between men and women, as well as influences related to hormone levels, genetics, and other factors. However, a study from Sweden found no difference in the prevalence of cognitive impairment between genders [46]. The discrepancy between this result and our findings may stem from differences in the scales used to measure cognitive function, as well as potential sociodemographic variations such as ethnicity and geography. A study by Salk and colleagues also indicates that women exhibit a higher level of severe depressive symptoms compared to men [47]. Depression has become a stereotyped illness for women, which may lead to an over diagnosis of depression in women. Conversely, this stereotype may result in the neglect of depression in men. Our results also show that the combination of declining cognitive function and increasing depressive symptoms is more common among older participants with low grip strength and shorter sleep duration. The factors influencing cognitive function cover multiple aspects such as demographic and socioeconomic factors, lifestyle, and health behaviours [48]. As individuals age, the reduction in the number of neurons and the thinning of the cerebral cortex, along with decreased neurotransmitter synthesis and reduced connections between neurons, lead to a decline in brain neural network function, thereby affecting cognitive abilities. Additionally, older adults may experience decreased social activities, lack of exercise, and an increased incidence of chronic diseases, which may also accelerate the decline in cognitive function.

#### The relationship between sleep duration, cognitive and depression trajectories

Our analysis provided evidence that sleep duration was associated with cognitive trajectory, depression trajectory and dual trajectories. Sleep duration has an inverted U-shaped relationship with cognitive trajectory, while depression trajectory and dual trajectories have a U-shaped relationship. Sleep duration has an inverted U-shaped relationship with cognitive trajectory, while depression trajectory and dual trajectories have a U-shaped relationship. This indicates that moderate sleep helps maintain a stable state of good cognition and low depression. In previous studies, sleep duration of six hours or less was associated with lower memory performance, while sleep durations of nine hours or more were associated with poorer performance on executive function tests [10]. A study summarising the English Longitudinal Study of Ageing (ELSA) and CHARLS cohorts also found an inverted U-shaped association between sleep duration and overall cognitive decline [8], which supports our conclusion. Another study using data from the UK Biobank for participants primarily of European ancestry aged 38–73 years, identified a nonlinear relationship between sleep duration and mental health and cognitive and determined that approximately seven hours is the optimal sleep duration [49]. Similarly, the National Health and Nutrition Examination Survey (NHANES) data revealed a U-shaped relationship between depression and sleep duration among American adults. The risk of depression was inversely related to sleep duration, reaching the lowest point at eight hours [13]. However, when sleep duration exceeds eight hours, the risk of depression significantly increases. Although these studies have slightly different sleep duration thresholds compared to our research (possibly due to different measurement methods or racial differences), the trends are consistent with our conclusions.

Possible mechanisms include abnormal melatonin secretion and disruptions in the sleep-wake cycle, which can affect sleep rhythms and potentially trigger conditions such as depression and cognitive impairment [50]. Additionally, poor sleep leads to fatigue, impairs hippocampal activity, alters immune responses, and affects the levels of pro-inflammatory and anti-inflammatory molecules. These factors can increase neuroinflammation and microglial activation, leading to cognitive dysfunction and depression [51]. Furthermore, prolonged sleep is significantly associated with memory deficits, and memory may change due to alterations in sleep habits [8], potentially leading to more severe symptoms of depression and a decline in cognitive abilities [10,52,53]. These findings are significant for long-term self-adjustment of daily behaviours to prevent cognitive dysfunction and reduce the risk of depression in older adults.

#### The relationship between grip strength, cognitive and depression trajectories

We found grip strength was nonlinearly associated with different trajectories. Grip strength is now broadly recognised and validated as a measure of general health, and it is progressively regarded as a predictor of mental health risks and neurodegenerative conditions in the elderly [54]. Our research has revealed that robust grip strength is correlated with a favourable cognitive trajectory, low depression trajectory and stable state. This is similar to the findings of Jiang et al. [54], who analysed data from over 40 000 participants in the UK Biobank and examined the complex interactions between grip strength, behavioural outcomes, and brain structure. They suggested that grey matter volume largely mediates the relationship between grip strength and cognitive and mental health. These findings indicate that grip strength can serve as a malleable indicator for detecting early damage in specific regions.

Notably, our study employed a longitudinal approach, delving deeper into the continuous development of changes rather than a simple analysis at a single time point, helping us to understand more deeply and comprehensively the relationship between grip strength and cognitive and depression. The potential mechanism of the relationship between grip strength and depression trajectory may be mediated by the influence of specific brain area volume, C-reactive protein, neutrophils, and white blood cells [55]. In addition, brain-derived neurotrophic factor, as a nutritional factor, plays a crucial role in exercise interventions [56]. Grip strength is a reflection of the integrity of the nervous system. The cortical and subcortical regions involved in hand strength and gripping activities control cognitive functions, influencing cognition by stimulating the prefrontal cortex and hippocampus [57,58].

#### The mediating role of sleep duration and grip strength between ADLs and different trajectories

Further, our study discovered that sleep duration and grip strength partially mediate the association between ADLs and different trajectories. Previous studies have shown correlations between sleep duration, grip strength, and ADLs with cognition or depression [15,54,59], but did not find that sleep duration and grip strength partially mediated the relationship between ADLs and these trajectories. Although sleep duration and grip strength do not play a dominant role in mediating the association between ADLs and the various trajectories, there may be other mediating variables such as brain function and grey matter volume [54]. However, it still suggests that maintaining appropriate sleep time and good grip strength in middle-aged and elderly people can help prevent the risk of cognitive decline and depression.

### Public health implication

The strength of our study lies in the use of a large, representative sample database for population-based, longitudinal research. Conducting an eight-year follow-up examination of depressive symptoms and cognitive function among the sample population allows us to understand more systematically and thoroughly the trajectories of cognition and depression, as well as their dual trajectories change and related influencing factors. This approach provides a new perspective on the complex relationship between cognitive function and depression. Long-term tracking and more in-depth research exploring these associations have guiding significance for developing effective prevention strategies for the cognitive and mental health of middle-aged and elderly people, which can help reduce the economic burden of medical care and improve their quality of life.

According to our research, we suggest that middle-aged and elderly adults keep sleeping for around seven hours to achieve a more stable cognitive and depression state. The forearm muscles play a crucial role in grip strength and ADLs, and effective enhancement of this strength can be achieved through exercises such as wrist extension, wrist flexion, wrist rotation, resistance training, and blood flow restriction training [60–63]. By identifying high-risk individuals and modifiable risk factors, targeted interventions can be taken to provide quality services for vulnerable populations, aiming to reduce the risk of cognitive decline and depression.

### Limitations of the present work

However, our study still has some limitations. First, we relied on the observational data from the CHARLS, where potential recall and self-reported bias likely existed. Although we strive to control for various common potential confounding factors related to cognitive function and depression, it is important to recognise that there may be other residual and unmeasured confounders that have not been considered but can affect disease outcomes, such as genetic factors and dietary structure, which were not available in our study. Second, although our study found that sleep duration and grip strength partially mediate the association between ADLs and cognitive trajectory, depression trajectory, and dual trajectories, they do not dominate the mediation of the association between ADLs and the various trajectories. Further research is needed to explore more mediating factors such as moderate exercise, social activity, and diet. Third, our study focused on the associations between baseline sleep time, grip strength and different trajectories of cognition and depression. The temporal relationship was clear, but the possibility of reverse causality cannot be ruled out. More longitudinal data needs to be collected to verify this reverse causality. Finally, our research primarily focuses on data from the middle-aged and elderly population in China, which may not fully capture the global scenario. In future research, we may consider expanding the scope of our study to include more domestic and international databases, thereby enhancing the international representativeness of our findings.

## CONCLUSIONS

Our study demonstrated that sleep duration and grip strength are not only associated with cognitive, depression, and dual trajectories, but also partially mediate the relationship between ADLs and these trajectories. This study provides a scientific basis for promoting cognitive and mental health in middle-aged and elderly adults. Our findings suggest that strategies to prevent cognitive impairment and depression should focus on ensuring appropriate sleep duration, maintaining good grip strength, and encouraging middle-aged and elderly adults to engage in daily living exercises.

## Additional material


Online Supplementary Document
