# Supplementary material for: Longitudinal patterns of cognitive function and depression: insights from the China Health and Retirement Longitudinal Study
Source: J Glob Health. 2025 Feb 28;15:04060. doi: 10.7189/jogh.15.04060 (PMC11868976; doi:10.7189/jogh.15.04060)
Supplement: Online Supplementary Document [file jogh-15-04060-s001.pdf]

Supplementary File

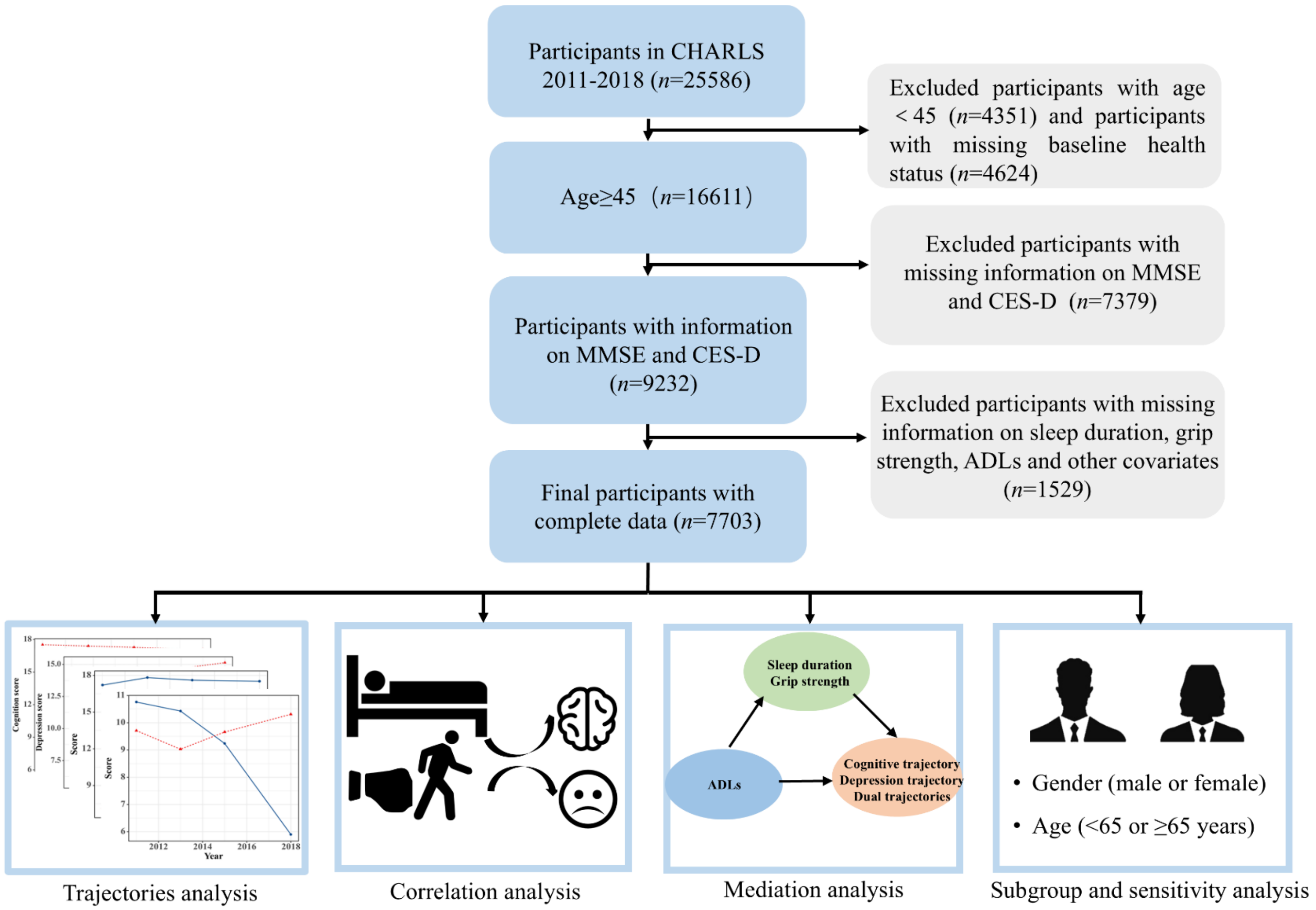

Figure S1. Flow chart of participant selection

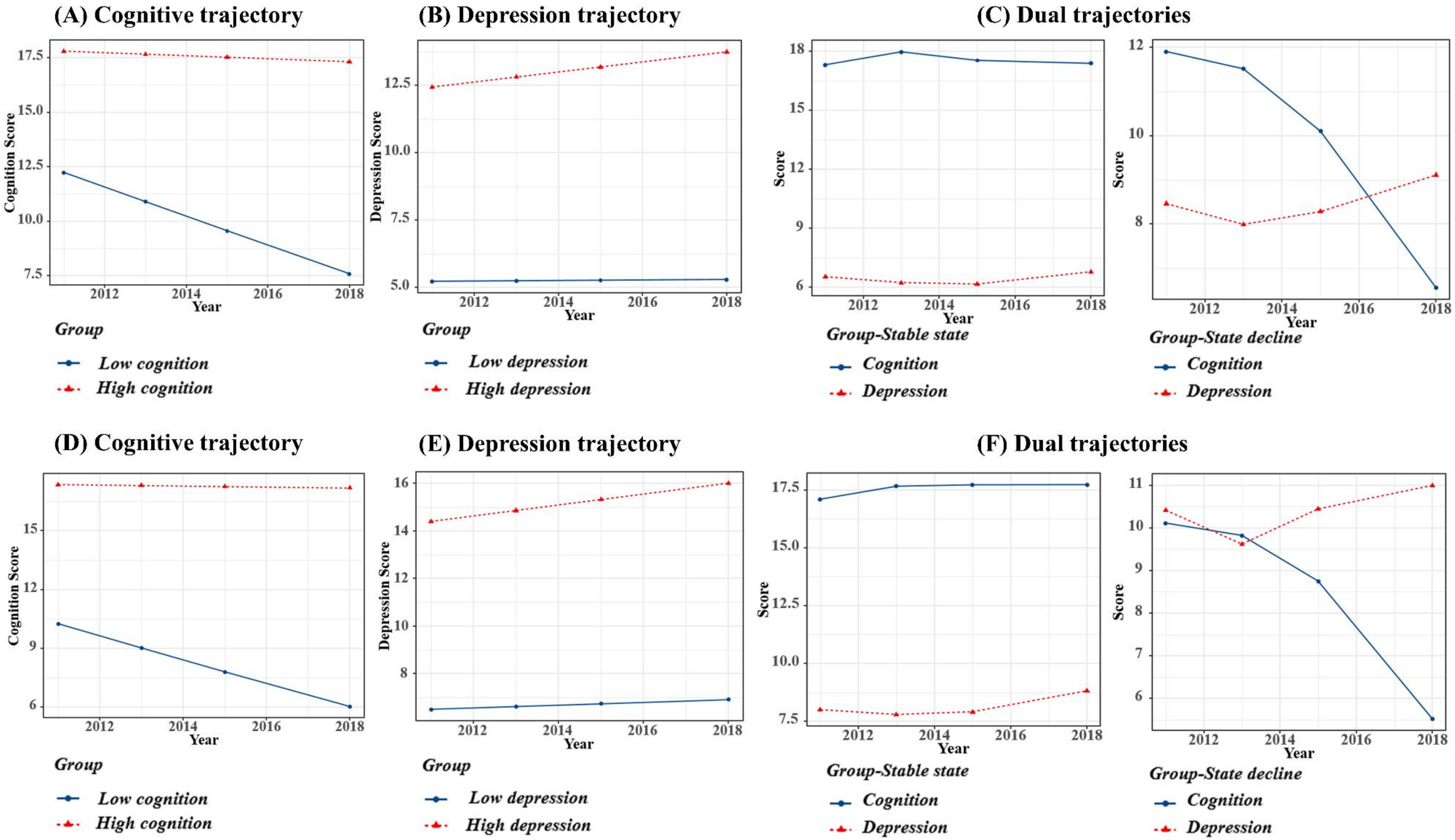

Figure S2. Cognition trajectory, depression trajectory, and dual trajectories of different gender groups. **Panel A-C.** Different trajectories of male participants; **Panel D-F.** Different trajectories of female participants.

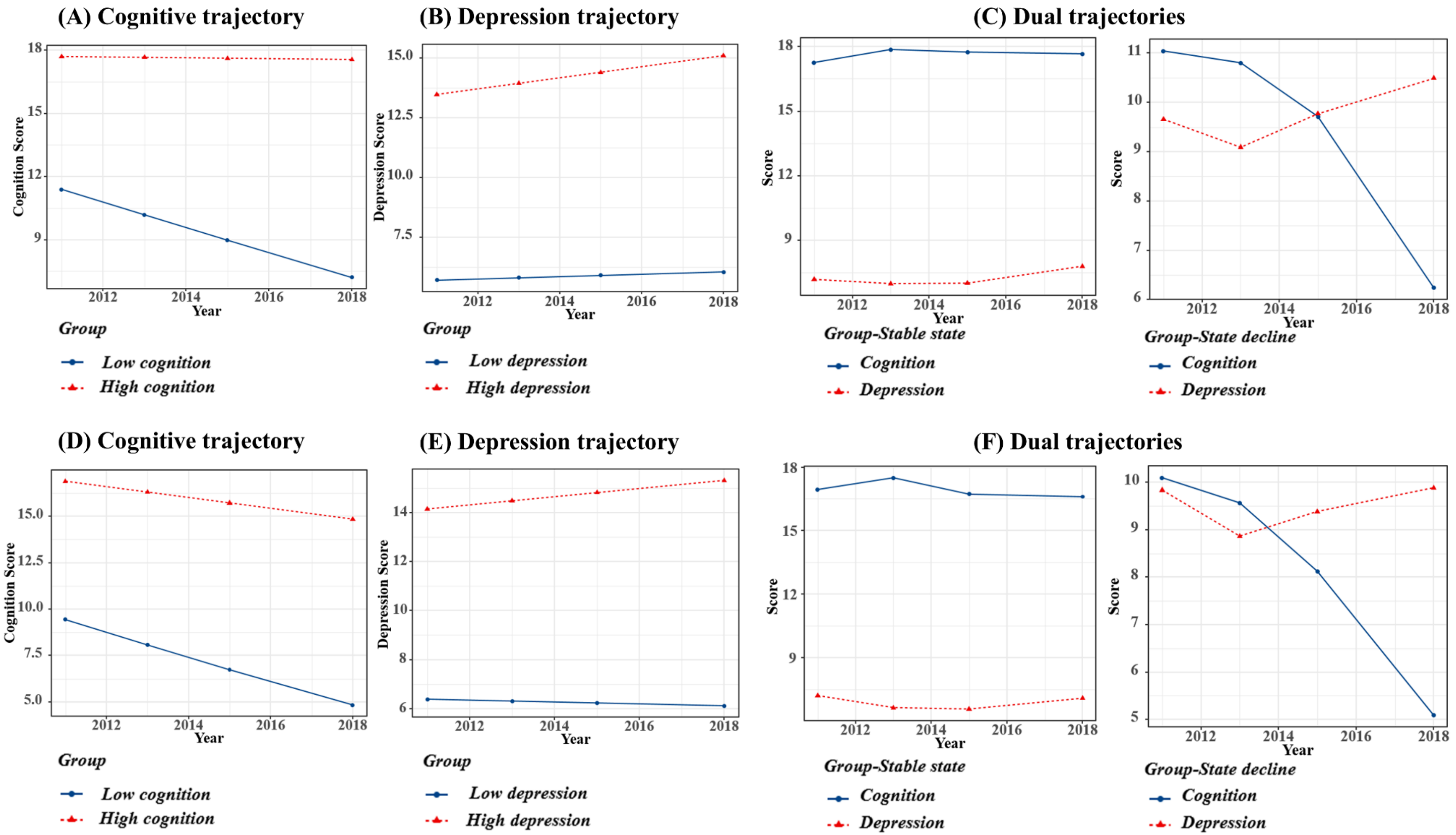

Figure S3. Cognition trajectory, depression trajectory, and dual trajectories of different age groups. **Panel A-C.** Different trajectories of participants <65 years old; **Panel D-F.** Different trajectories of participants ≥65 years old.

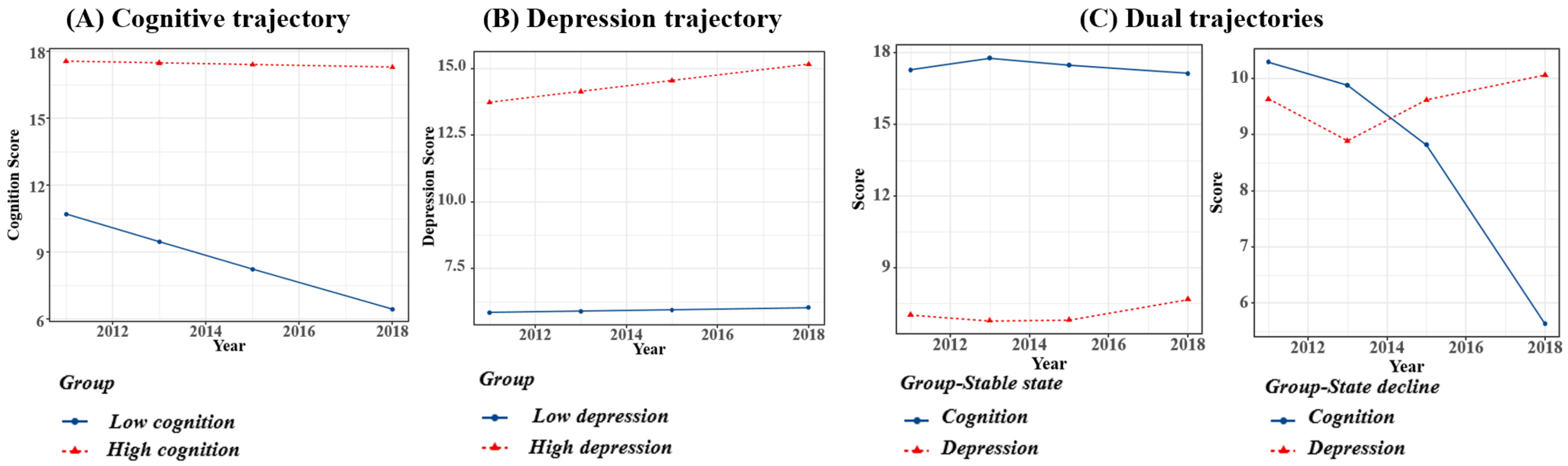

Figure S4. Cognition trajectory, depression trajectory, and dual trajectories of cognition and depression in sensitivity analysis.

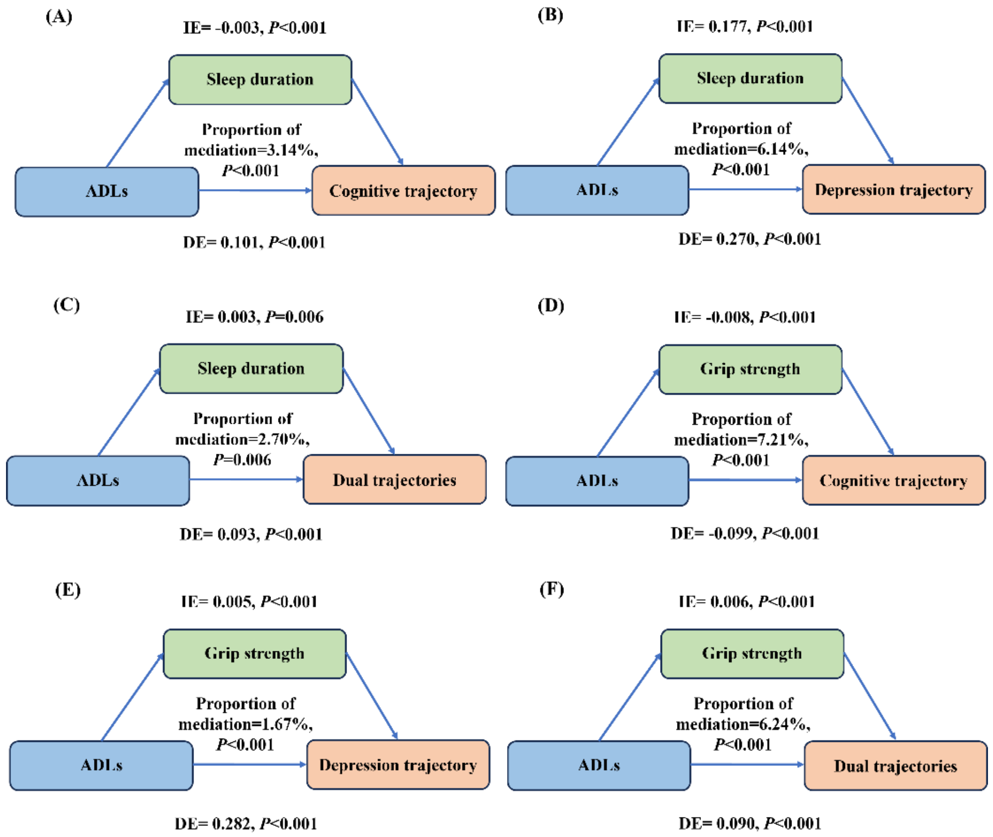

Figure S5. The mediating effects of sleep duration and grip strength on ADLs and different trajectories. **Panel A**. The mediating role of sleep duration on ADLs and cognitive trajectory. **Panel B.** The mediating effect of sleep duration on ADLs and depression trajectory. **Panel C.** The mediating effect of sleep duration between ADLs and dual trajectories. **Panel D**. The mediating effect of grip strength on ADLs and cognitive trajectory. **Panel E**. The mediating effect of grip strength on ADLs and depression trajectory. **Panel F**. The mediating effect of grip strength between ADLs and dual trajectories. ADLs – activities of daily living.
